# Residual Fistula of Fourth Branchial Arch Anomalies and Recurrent Left-Side Cervical Abscess: Clinical Case and Review of the Literature

**DOI:** 10.1155/2014/931279

**Published:** 2014-11-24

**Authors:** Bassel Hallak, Salim Bouayed, Crispin Leishman, Kishore Sandu

**Affiliations:** Department of Otorhinolaryngology, Head and Neck Surgery, Hospital of Sion, 1950 Sion, Switzerland

## Abstract

Congenital fourth branchial arch anomalies are uncommon entities. Most of these anomalies are diagnosed in childhood. The majority of cases occur on the left side. The clinical presentation of these anomalies varies with age. A respiratory distress is the usual clinical presentation in neonates, cervical cutaneous fistulas in late childhood or acute suppurative thyroiditis. Multiples diagnostic options have been described with different modalities of treatment. The majority of cases of fourth branchial arch anomalies are described only in case reports. We report a clinical case of recurrent cervical abscess in a young woman due to a residual fistula of fourth branchial arch.

## 1. Introduction

The branchial arches give rise to specific derivatives, which for the fourth arch include the laryngeal cartilages, the laryngeal and pharyngeal muscles, the superior laryngeal nerve, the left thoracic aorta, the right proximal subclavian artery, the interfollicular cells of the thyroid, and the superior parathyroid glands [1].

Congenital fourth branchial arch anomalies are uncommon entities. They typically present as a cervical inflammatory process primarily affecting children. Acute suppurative thyroiditis is a common presentation for this pathology (45%). Almost all cases of these anomalies are described only in case reports (526 cases were found in the literature) [2].

Multiple diagnostic and treatment options have been described with a large variety in outcomes.

The first published description of a branchial cleft lesion was in 1832 by Ascherson [3]. The prevalence of fourth arch anomalies is 1% to 4% of all branchial anomalies [4].

We report a clinical case of a recurrent left-side cervical abscess in a young woman due to a residual fistula between the pyriform sinus and the thyroid lobule on the left side. She had been operated for an excision of a cervical cyst in the left side 6 years before with an initial diagnosis of a fourth branchial anomaly.

## 2. Clinical Case

**Figure 1:**
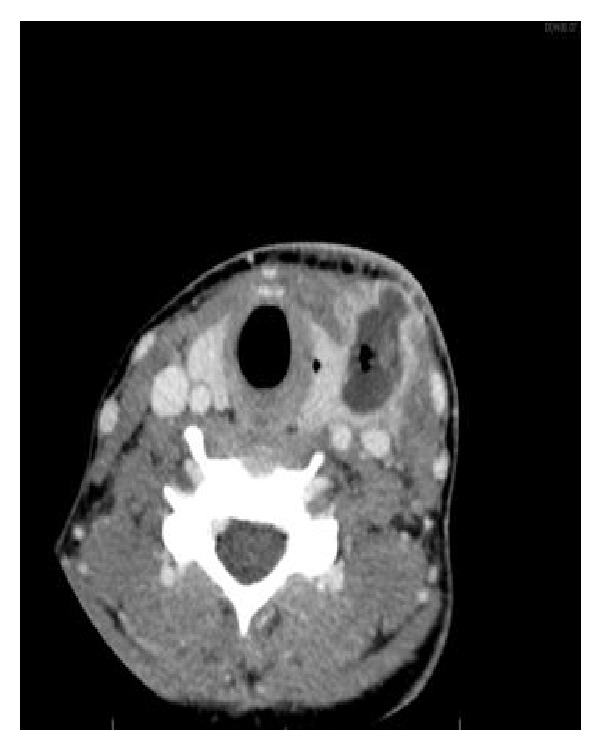
Axial view CT-scan.

**Figure 2:**
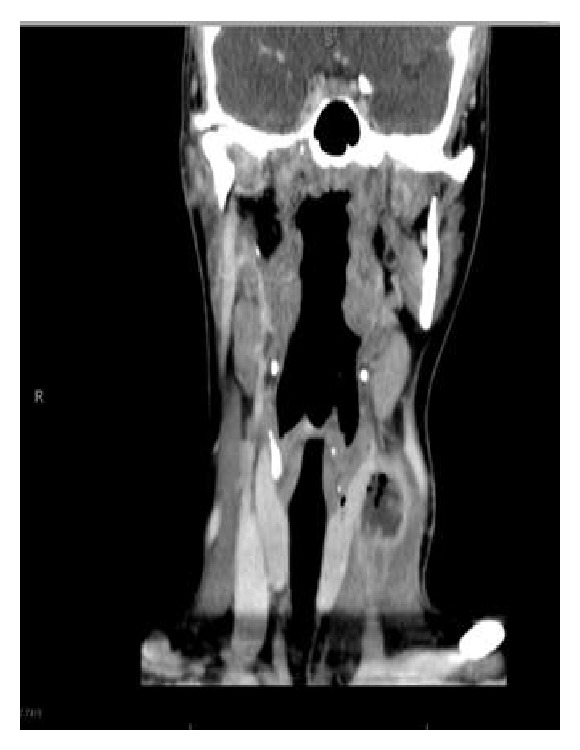
Coronal view CT-scan.

A 20-year-old woman presented in January 2013 with an inflammatory left-side cervical swelling. She had been in a good general condition and only experienced an excision of a left-side cervical cyst 6 years before with an initial diagnosis of a fourth branchial arch anomaly. The follow-up did not show any recurrence or inflammatory process during the last 6 years. The clinical examination showed a left-side inflammatory and painful cervical swelling without evidence of external skin fistula or spontaneous discharge of pus. She had no dyspnea or dysphagia. The ENT examination did not show any pathological signs in the oropharynx, hypopharynx, or larynx. The biological findings showed an acute inflammatory syndrome with leukocytosis and elevated CRP. A cervical CT-scan showed a multilobulated liquid formation in the left lateral cervical region, measuring 29 × 15 × 55 mm, in contact with the left thyroid lobule, as well as the presence of a fistula tract between the thyroid lobule and the pyriform sinus on the left side (Figures 1 and 2).

**Figure 3:**
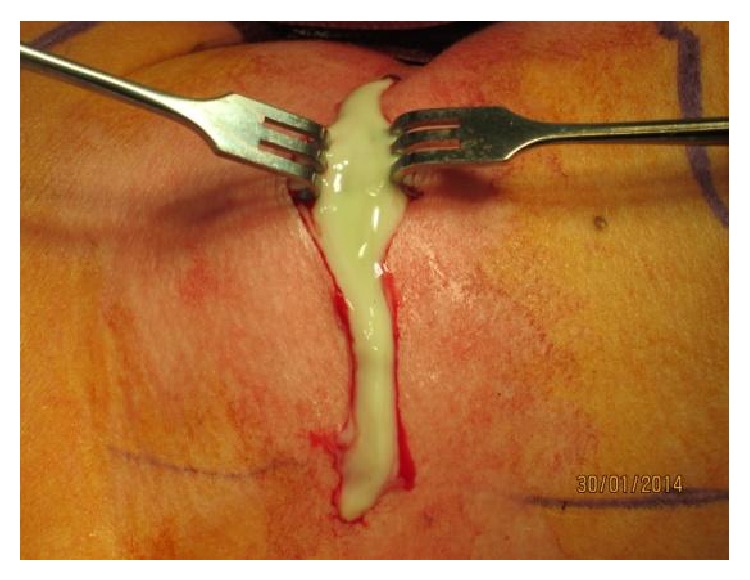
Preoperative view.

A surgical drainage of the abscess was done, followed by an antibiotic treatment of amoxicillin-clavulanic acid with a dose of 1 gr three times per day for 14 days (Figure 3).

**Figure 4:**
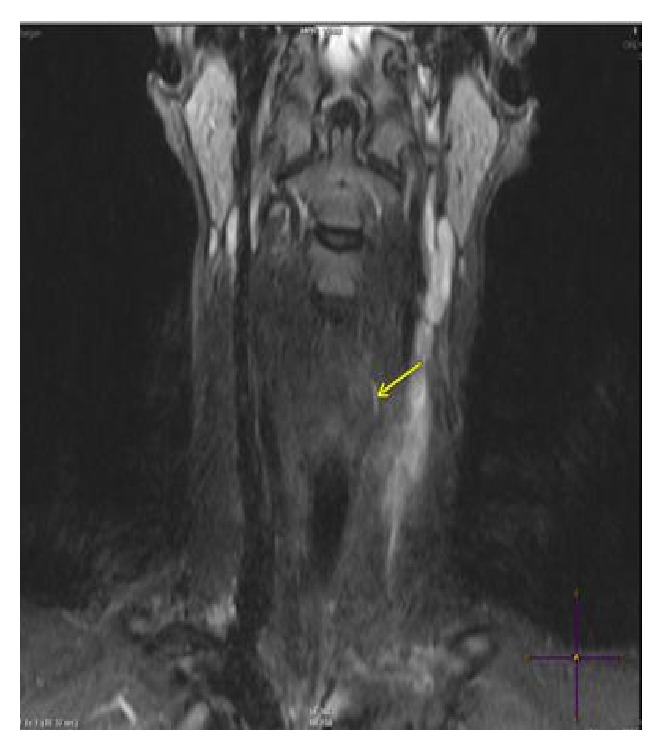
T1 MRI coronal view after injection.

The 2-month postoperative follow-up showed good healing of the cervical wound without any signs of recurrence. An MRI was done 3 months after the surgery and showed a diffuse inflammation in the left cervical region without abscess formation and a fistula tract at the level of the hypopharynx on the left side (Figure 4).

**Figure 5:**
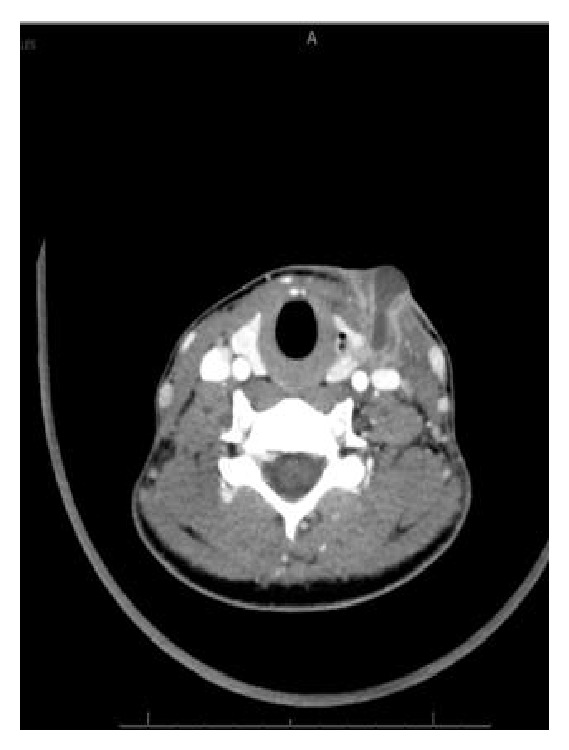
Axial CT-scan.

A complete surgical treatment including the resection of the fistula tract and left thyroid lobule combined with endoscopic control of the fistula in the left pyriform sinus was proposed to the patient; unfortunately, she refused the surgery at that time. Five months later, she presented a recurrence of a cervical abscess on the left side. A cervical CT-scan showed an abscess with a fistula on the left side of the neck (Figure 5). A second surgical drainage was done, followed by an antibiotic therapy for a period of 14 days.

**Figure 6:**
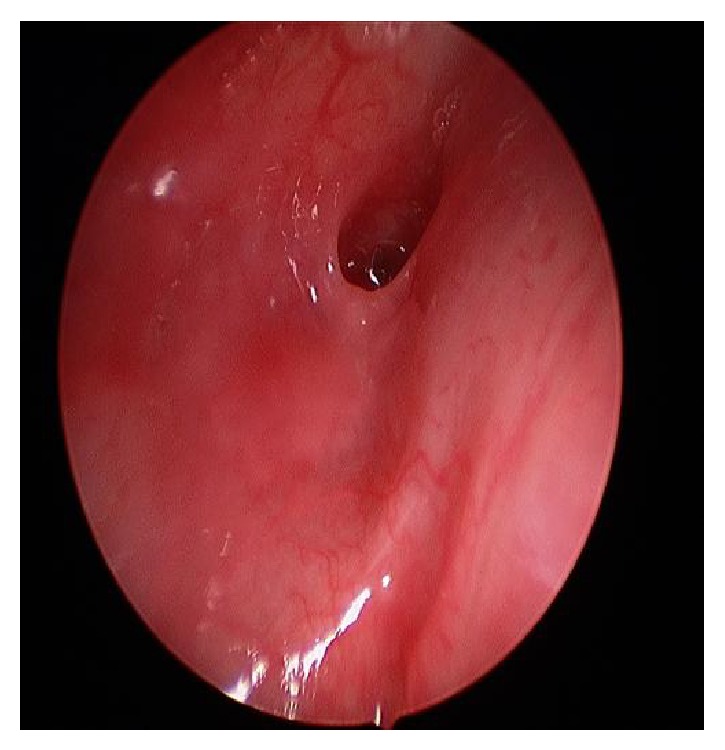
Left piriform sinus.

**Figure 7:**
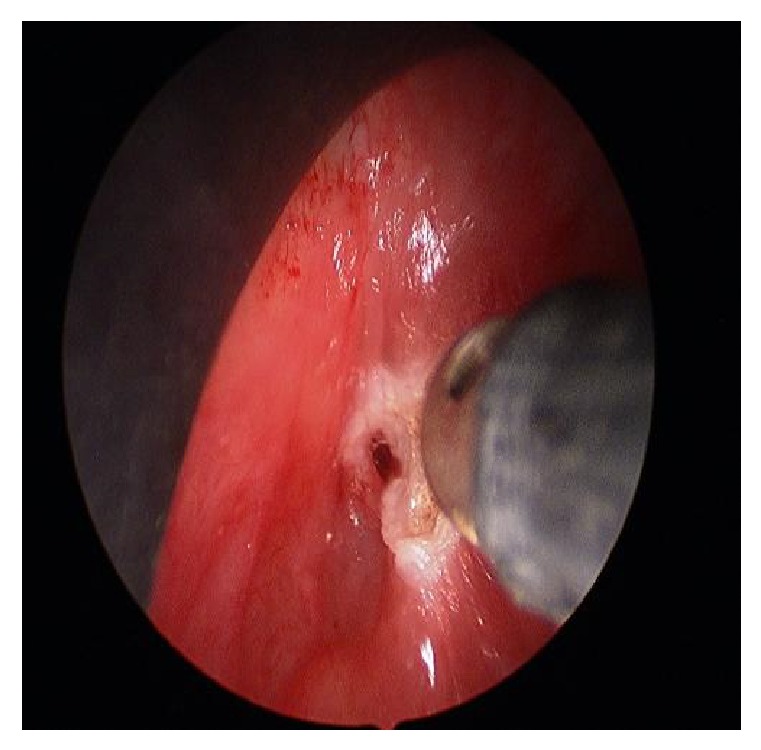
Cauterization of the orifice.

**Figure 8:**
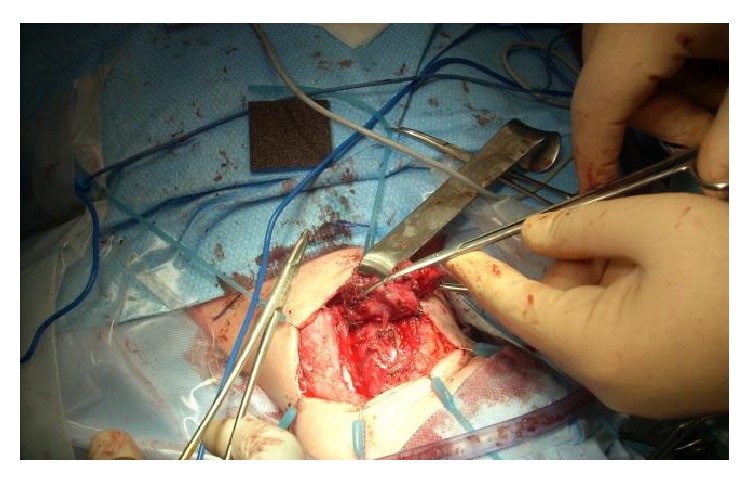
Intraoperative view.

A complete surgical treatment was performed 2 months later. The intraoperative endoscopy showed a fistula orifice in the apex of the left piriform sinus. An endoscopic cauterization of the fistula in the left piriform sinus was done (Figures 6 and 7). A left-side cervicotomy was performed with a complete excision of the fistula tract, left thyroid lobule, and all the inflammatory tissues with multiples lymph nodes (Figure 8: fistula's tracts between the left thyroid nodule and the pharynx). The surgery was followed by an antibiotic treatment. The postoperative follow-up showed a good healing of the wound, no symptoms of thyroid gland dysfunctions or any other complications.

## 3. Discussion

Most fourth branchial arch anomalies are diagnosed in childhood. The great majority of cases occur on the left side and most commonly present either as acute suppurative thyroiditis or neck abscess. Respiratory distress could be a clinical presentation for these anomalies in neonates. In the 518 cases found in the literature in which the patient's age was reported, 45 occurred in neonates and 29 (64%) of these presented with respiratory distress [2].

Fourth branchial arch anomalies represent vestiges of a sinus tract, originating from the apex of the pyriform sinus [5]. Some authors group third and fourth arch fistulas generically as pyriform sinus tracts or pyriform fossa lesions [6, 7]. But anatomically, these two types of anomalies are distinct: the tract of the third branchial arch anomalies originates from the base of the pyriform sinus and the tract of the fourth branchial arch anomalies originates from the apex of the pyriform sinus [5].

The clinical presentation of fourth branchial arch anomalies varies with age. A respiratory distress is the usual clinical presentation in neonates. Cervical cutaneous fistulas develop only in late childhood. Acute suppurative thyroiditis is a late clinical presentation. Cases of mediastinal abscess of fourth branchial arch origin have been reported [8, 9].

The left side predominance of the disease might be because of the more complex and extended pathway of the fourth branchial tract on the left side [10].

In a meta-analysis of 526 cases published in a literature, the fourth branchial arch anomalies were almost always located on the left side (94%) and generally presented as acute suppurative thyroiditis (45%) or recurrent neck abscess (42%) [2].

A large variety of diagnostic procedures have been described. The barium esophagogram may fail to demonstrate a sinus tract during acute inflammation [4]. In contrast, direct laryngoscopy can be performed during an acute episode and can achieve several targets [11]. Magnetic resonance imaging is especially useful for detecting cystic anomalies of the sinus tract. The use of Valsalva maneuver during CT-scan or barium swallow may help to open the sinus tract and make it more readily visible [12, 13]. The meta-analysis of 526 cases showed that the barium esophagogram and direct laryngoscopy were the most useful diagnostic tools (74% and 45% of cases) [2].

The treatment options are multiple. Incision and drainage is frequently performed but carries a high recurrence rate. By contrast, open neck surgery, especially when combined with partial thyroidectomy, has the lowest recurrence rate but yields a higher rate of complications in infants and small children [2], with more risk of injury to the cervical neurovascular structures related to the small neck in this age. Endoscopic treatment represents a minimally invasive technique using cauterization to obliterate the internal opening of a pyriform sinus tract [14]. The meta-analysis of 526 cases described recurrence rates: incision-drainage, 89%; open neck surgery and tract excision, 15%; endoscopic cauterization of the sinus tract opening, 15%; and open neck surgery with partial thyroidectomy, 8% [2].

In our case, the 2 episodes of acute inflammation presented as left sided neck abscess without signs of thyroiditis and were treated by incision-drainage followed by antibiotic therapy. After the acute phase, open neck surgery with complete excision of the fistula's tract combined with left side partial thyroidectomy and cauterization of the internal opening of the left pyriform sinus tract by endoscopic approach were performed. No complications or recurrence of inflammation was noted in the postoperative follow-up. The patient had no difficulties with swallowing. A postoperative indirect laryngoscopy showed normal vocal cord mobility and absence of edema in the pyriform sinus. We noted good healing of the cervical wound.

## 4. Conclusion

Fourth branchial arch anomalies are more common than previously suspected. The usual clinical presentations of these anomalies are recurrent neck abscess, acute suppurative thyroiditis, and respiratory distress in neonatal period. Complete excision of the fistula tract during a quiescent period combined with partial thyroidectomy appears to be the treatment of choice with a lower recurrence rate. This surgery is recommended for patients older than age 8 to minimize complications. Before the age of 8, a medical treatment is recommended. Endoscopic cauterization can be an effective alternative strategy with an outcome similar to open surgery.
